# Multimorbidity and emergency hospitalisations during hot weather

**DOI:** 10.1016/j.ebiom.2024.105148

**Published:** 2024-05-04

**Authors:** Zhiwei Xu, Weizhuo Yi, Aaron Bach, Shilu Tong, Kristie L. Ebi, Hong Su, Jian Cheng, Shannon Rutherford

**Affiliations:** a School of Medicine and Dentistry, Griffith University, Gold Coast, Australia; b Cities Research Institute, Griffith University, Gold Coast, Australia; c School of Public Health, Anhui Medical University, Hefei, China; d National Institute of Environmental Health, Chinese Center for Disease Control and Prevention, Beijing, China; e School of Public Health and Social Work, Queensland University of Technology, Brisbane, Australia; f Center for Health and the Global Environment, University of Washington, Seattle, USA

**Keywords:** Chronic disease, Heat-health action plan, Multimorbidity

## Abstract

**Background:**

People with chronic diseases are a commonly listed heat-vulnerable group in heat-health action plans. While prior research identifies multiple health conditions that may increase vulnerability to ambient heat, there is minimal evidence regarding the implications of multimorbidity (two or more chronic diseases).

**Methods:**

From the statewide hospital registry of Queensland, Australia, we identified people aged ≥15 years who had emergency hospitalisation(s) between March 2004 and April 2016 and previously had 0, 1, 2, or ≥3 of five chronic diseases: cardiovascular disease, diabetes, mental disorders, asthma/COPD, and chronic kidney disease. We conducted time-stratified case-crossover analyses to estimate the odds ratio of hospitalisations associated with ambient heat exposure in people with different numbers, types, and combinations of chronic diseases. Ambient heat exposure was defined as a 5 °C increase in daily mean temperature above the median.

**Findings:**

There were 2,263,427 emergency hospitalisations recorded (48.7% in males and 51.3% in females). When the mean temperature increased, hospitalisation odds increased with chronic disease number, particularly in older persons (≥65 years), males, and non-indigenous people. For instance, in older persons with 0, 1, 2, or ≥3 chronic diseases, the odds ratios associated with ambient heat exposure were 1.00 (95% confidence interval: 0.96, 1.04), 1.06 (1.02, 1.09), 1.08 (1.02, 1.14), and 1.13 (1.07, 1.19), respectively. Among the chronic diseases, chronic kidney disease, and asthma/COPD, either existing alone, together, or in combination with other diseases, were associated with the highest odds of hospitalisations under ambient heat exposure.

**Interpretation:**

While individuals with multimorbidity are considered in heat-health action plans, this study suggests the need to consider specifically examining them as a distinct and vulnerable subgroup.

**Funding:**

10.13039/100004440Wellcome.

**Research in context**

**Evidence before this study**

We searched PubMed, Scopus, and Web of Science to retrieve articles published in English from January 1, 2000, to October 1, 2023. Compared with individuals without chronic diseases, people with pre-existing chronic diseases have a higher risk of morbidity and mortality under ambient heat exposure (i.e., exposure to high ambient temperatures). Although multimorbidity (i.e., the co-existence of two or more chronic diseases) is prevalent worldwide (51.0% in those >60 years), we did not find published evidence on the association between the number of pre-existing chronic diseases and the risk of morbidity and mortality under ambient heat exposure.

**Added value of this study**

Under ambient heat exposure, the odds of hospitalisations increase with the number of pre-existing chronic diseases, particularly in older persons (≥65 years), males, and non-indigenous people. Among different types of pre-existing chronic diseases, chronic kidney disease, and asthma/COPD, either existing alone, together, or in combination with other diseases, are associated with the highest odds of hospitalisations under ambient heat exposure.

**Implications of all the available evidence**

Individuals with a greater number of chronic diseases face a higher risk of hospitalisations under ambient heat exposure. There is a need to consider examining people with multimorbidity as a distinct and vulnerable subgroup in heat-health action plans.

## Introduction

Exposure to hot weather is detrimental to human health and well-being.^1^ Effective heat-health action plans (HHAPs, early warnings of impending extreme heat events [i.e., heatwaves] accompanied by a range of heat-intervention actions) can protect the health of the public from the adverse impacts of heat.^2^ Although there is no universally consistent definition of heatwaves, heatwaves are often defined as prolonged days of extreme heat when ambient temperature exceeds certain thresholds.^3^ The heat-intervention actions, such as postponing sporting activities and working with utilities to prevent suspensions of water and electricity service,^4^ mainly aim at reducing heat exposure and/or improving access to and use of cooling measures.

An important step to effectively implementing an HHAP is to make sure that heat-health risk information is delivered and targeted to heat-vulnerable subgroups.^5^ People with pre-existing heat-sensitive chronic diseases (e.g., cardiovascular disease) have been widely recognised as a heat-vulnerable subgroup in HHAPs.^5^ However, these chronic diseases are often not suffered in isolation. For instance, an underlying cause of chronic kidney disease is hypertension.^6^ It is plausible that people with two chronic diseases (e.g., cardiovascular disease plus chronic kidney disease) are more vulnerable to heat exposure than those with a single chronic disease (e.g., cardiovascular disease). Although the co-existence of two or more chronic diseases (i.e., multimorbidity) is prevalent worldwide (37.2% in people ≥18 years and 51.0% in those >60 years^7^), there is limited epidemiological or physiological evidence of the association between multimorbidity and heat-health risk.^8^ Generating this evidence is key to nuanced targeting of heat-vulnerable subgroups in the face of heatwaves as part of HHAPs.

Aside from delivering heat-health risk information to heat-vulnerable subgroups, alerting hospitals and other health services about the potential elevation in patient volume before heatwaves is another important step in implementing an HHAP.^5^ This is because these health services may need to take preparedness measures (e.g., deploy extra staff) before the onset of heatwaves to improve operational efficiency and save lives. From a clinical perspective, patients with multimorbidity typically require more comprehensive and integrated medical management than patients with one or no chronic disease.^9^ Therefore, it is useful to investigate the association between ambient heat exposure and the odds of cause-specific hospitalisations in people with multimorbidity.

This study aimed to bridge the knowledge gap by (1) examining the association between ambient heat exposure (a 5 °C increase in daily mean temperature above the median) and the odds of all-cause hospitalisations in people with 0, 1, 2, or ≥3 chronic diseases; (2) identifying the chronic disease types and combinations that were associated with the highest odds and burden of hospitalisations under ambient heat exposure; and (3) quantifying the association between ambient heat exposure and the odds of cause-specific hospitalisations in people with multimorbidity.

## Methods

### Study population

In this study, we used data from the statewide hospital registry of the Queensland State, Australia: Queensland Hospital Admitted Patient Data Collection (QHAPDC). Queensland is the 3rd most populous state in Australia and home to more than 5 million people (according to the 2021 Australian Census data^10^), and it is one of the several regions worldwide predicted to be most at risk of record-breaking heatwaves in the coming decades.^11^ QHAPDC collects demographic and clinical information on all patients admitted to public or licensed private hospitals in Queensland. The original data was routinely collected by each hospital and was then reported to the Statistical Services Branch (SSB) at Queensland Health. Data validation was conducted by each hospital prior to submission to SSB. More detailed information of QHAPDC, such as hospitals included in the QHAPDC and data validation process, can be found here: https://www.health.qld.gov.au/hsu/collections/qhapdc. People from eight Queensland cities/communities who had emergency hospitalisations (hereinafter called ‘hospitalisations’ for simplicity) between 1st March 2004 and 30th April 2016 (the date of admission) were included in the analysis. The eight cities/communities included Brisbane, Townsville, Cairns, Toowoomba, Mackay, Rockhampton, Mount Isa, and Longreach (Figure S1; Appendix), accounting for more than 60% of the Queensland population (according to the 2021 Australian Census data^10^).

### Ethics

Ethical approval (approval number: 1500000369) was granted by the Queensland University of Technology Human Research Ethics Committee before the data collection.

### Measurement of temperature exposure

The QHAPDC data included each patient's postcode information, and we used daily postcode-level temperature to represent their daily ambient temperature exposure. The daily postcode-level temperature data including maximum and minimum temperatures were sourced from the Australian Bureau of Meteorology online archive.^12^ We calculated the daily postcode-level mean temperature by averaging maximum and minimum temperatures because our prior works found mean temperature to be slightly more predictive of heat-related mortality and morbidity.^13^^,^^14^

### Sociodemographic variables

The QHAPDC data included each patient's information on age, sex, and indigenous status. Similar to the Australian Burden of Disease Study, the QHAPDC data categorised people's age into several groups (<1, 1–4, 5–14, 15–24, 25–34, 35–44, 45–54, 55–64, and ≥65 years).^15^ We included those aged ≥15 years in this study because chronic disease is less prevalent in those aged 0–14 years. We categorised people aged ≥15 years into two groups: working-age individuals (15–64 years) and older persons (≥65 years). We categorised indigenous status into two groups: indigenous (including Aboriginals, and Torres Strait Islanders), and non-indigenous. We obtained postcode-level Socio-Economic Indexes for Areas (SEIFA) data from the Australian Bureau of Statistics. SEIFA ranks the socioeconomic status of each area in Australia based on socioeconomic status.^16^ We used the Index of Relative Socioeconomic Disadvantage (IRSD) of SEIFA for each hospitalisation.^17^ Based on the percentile of IRSD, we categorised the socioeconomic status into three groups: high socioeconomic status (≤33rd percentile), middle socioeconomic status (34th–66th percentile), and low socioeconomic status (≥67th percentile) (i.e., a higher percentile of IRSD corresponded to a lower socioeconomic status).

### Ascertainment of pre-existing chronic diseases and primary causes of hospitalisations

For each hospitalisation, the QHAPDC data included the primary diagnosis and up to 10 other diagnoses at discharge coded using the International Classification of Diseases, Tenth Revision (ICD-10). We ascertained each participant's pre-existing chronic disease status at each hospitalisation based on both ‘the primary diagnosis and other diagnoses of all their previous hospitalisations’ and ‘the other diagnoses of their current hospitalisation’. We included five groups of chronic diseases in the calculation of chronic disease number, types, and combinations because these five groups of disease have been consistently reported to be associated with people's heat-health risk^5^^,^^8^: i) cardiovascular disease (hypertensive heart disease, ischaemic heart disease, heart failure, stroke); ii) diabetes; iii) mental disorders (schizophrenia, schizotypal and delusional disorders, bipolar disorders, depressive disorders, anxiety disorders, dementia); iv) asthma/COPD; and v) chronic kidney disease (detailed ICD-10 codes for each chronic disease presented in Table S1 [Appendix]). Based on our existing work on multimorbidity,^18^ we considered people with one of the five groups of chronic diseases at a hospitalisation as having one chronic disease at that hospitalisation. We acknowledged that some diseases (e.g., musculoskeletal disease, digestive disease) have been commonly used in calculating the number of diseases in multimorbidity research, but they are not very relevant in the heat and health context.

For quantifying the association between ambient heat exposure and the odds of cause-specific hospitalisations in people with multimorbidity (i.e., 2 or ≥3 chronic diseases), we chose the hospitalisations for heat-related illness (e.g., heat stroke) (ICD-10 codes: T67, E86, E87, X30^19^), infectious and parasitic disease (A00-B99), cardiovascular disease (I00–I99), mental disorders (F00–F99), respiratory disease (J00-J99), and urologic disease (N00–N99), because the published high level of evidence (i.e., systematic review and meta-analysis)^20^, ^21^, ^22^, ^23^, ^24^, ^25^ suggested that the likelihood of hospitalisations for these diseases increases under ambient heat exposure.

### Statistical analysis

To quantify the association between short-term exposure to ambient heat and the odds of hospitalisations, we used a time-stratified case-crossover design. A conditional logistic regression coupled with a distributed lag non-linear model was used to estimate the odds ratios and 95% confidence intervals of hospitalisations associated with ambient heat exposure. Following a common practice,^26^^,^^27^ we used a 5 °C increase in mean temperature as the increment to calculate the odds ratio associated with ambient heat exposure. We chose the 50th percentile of the daily mean temperature during the warm season (Nov, Dec, Jan, Feb, and Mar) of the study period as the reference temperature (25 °C). Aside from quantifying the association between short-term exposure to ambient heat and the odds of hospitalisations in the total population, we also conducted subgroup analyses by age, sex, indigenous status, and socioeconomic status.

The time-stratified case-crossover design has been extensively used to examine the association between short-term exposure to environmental hazards (e.g., ambient heat) and the risk of health events.^19^^,^^28^ This design has the advantage of automatically adjusting for known or unknown covariates that do not change from day to day (e.g., smoking, alcohol drinking behaviour). Hence, we did not further adjust for these covariates (e.g., age, sex) in the regression analyses. In the present study, the ambient heat exposure on the day when a hospitalisation occurred (case period) was compared with the ambient heat exposures on the same day of other weeks in the same month (control period). The analyses were based on hospitalisations (i.e., each hospitalisation as one case) and one patient could have had one or multiple hospitalisations. Because the existing epidemiological studies^29^^,^^30^ and our previous studies^31^^,^^32^ suggested that ambient heat exposure is not only associated with morbidity on the same day of exposure but also one to two days after exposure, we used a distributed lag non-linear model to capture the association lasting from the current day of exposure to two days after exposure (i.e., lag 0–2 days). We used a natural cubic spline with three degrees of freedom for the mean temperature to capture the potential non-linear relationship between mean temperature and hospitalisations.^33^ We did not adjust for other meteorological variables (e.g., rainfall) because of a lack of strong evidence on these variables being a potential confounder for the association between mean temperature and hospitalisations. After quantifying the odds ratio of hospitalisations associated with a 5 °C increase in mean temperature in hospitalisations with 0, 1, 2, or ≥3 chronic diseases, we conducted random effects meta-regression analyses to check if the effect estimates increased with the increase of chronic disease number.^34^

Apart from quantifying the odds ratio (OR) of hospitalisations associated with a 5 °C increase in mean temperature, we estimated the heat-attributable hospitalisation number by calculating hospitalisations attributable to all temperatures above the reference temperature (25 °C) (i.e., any temperature above 25 °C).^35^ Following our previous work,^34^ the attributable hospitalisations N_i_ on each day i were computed with the formula: N_i_ = D_i_ ∗ (OR_i_ - 1)/OR_i_, with D_i_ being the total hospitalisation number on day i. The total heat-attributable hospitalisation number was the sum of heat-attributable hospitalisations from all days when mean temperatures were above 25 °C. Following our previous work, if the association between ambient heat exposure and odds of hospitalisations was not statistically significant (95% confidence intervals covered 1), we assumed the heat-attributable hospitalisation number to be 0.^36^

### Sensitivity analysis

We conducted four sensitivity analyses to test the robustness of the main results: (1) the Heat-Health Action Plans: Guidance issued by the World Health Organization suggested that pre-existing Parkinson's disease is associated with heat-health risk,^5^ and hence we further included Parkinson's disease as the sixth group of chronic disease in the calculation of chronic disease number; (2) because some prior studies observed that heat exposure was associated with the increases in hospitalisations during the first few days but also the decreases in hospitalisations a few days later (i.e., harvesting effect),^37^^,^^38^ we adjusted the lag period from two days to a week to take into account the potential harvesting effect of ambient heat exposure. Harvesting effect refers to a reduction in hospitalisations at lags of several days after a hot day, because a pool of highly susceptible persons is depleted by the initial heat stress; (3) we used maximum temperature as the temperature indicator to test the robustness of the main results; and (4) we used 1, 2.5, and 7.5 °C increase in mean temperature as the increment to calculate the odds ratio associated with ambient heat exposure.

### Role of the funding source

The funder of the study had no role in study design, data collection, data analysis, data interpretation, or writing of the report.

## Results

### Sociodemographic factors and number of chronic diseases

There were a total of 2,263,427 hospitalisations with 0, 1, 2, or ≥3 chronic diseases in the eight cities/communities of Queensland during the study period (Table 1). As expected, older persons (≥65 years) had a higher proportion of 1, 2, or ≥3 chronic diseases (33.2%, 23.2%, and 15.0% respectively) than the working-age individuals (15–64 years) (25.5%, 7.6%, and 3.8% respectively). The proportion of ≥3 chronic diseases in males (8.5%) was slightly higher than that in females (7.5%), and the proportion of ≥3 chronic diseases in indigenous people (10.6%) was higher than that in non-indigenous people (7.8%). The proportion of ≥3 chronic diseases was higher in people with high socioeconomic status (9.5%), compared with those with middle socioeconomic status (7.6%) or low socioeconomic status (7.2%).

**Table 1:** Sociodemographic factors in people with 0, 1, 2, or ≥3 pre-existing chronic diseases when hospitalised.

	Number of pre-existing chronic diseases			
	0 (n = 1,137,061)	1 (n = 642,836)	2 (n = 303,464)	≥3 (n = 180,066)
**Age**				
15–64	895,875 (63.1%)	362,627 (25.5%)	108,032 (7.6%)	53,249 (3.8%)
≥65	241,186 (28.6%)	280,209 (33.2%)	195,432 (23.2%)	126,817 (15.0%)
**Sex**				
Male	538,886 (48.8%)	317,922 (28.8%)	153,065 (13.9%)	93,543 (8.5%)
Female	598,175 (51.6%)	324,913 (28.0%)	150,399 (13.0%)	86,523 (7.5%)
**Indigenous status**				
Non-indigenous	1,060,620 (50.3%)	601,450 (28.5%)	282,368 (13.4%)	163,634 (7.8%)
Indigenous	76,441 (49.2%)	41,386 (26.6%)	21,096 (13.6%)	16,432 (10.6%)
**Socioeconomic status**				
Low	450,280 (51.2%)	255,077 (29.0%)	111,299 (12.6%)	63,346 (7.2%)
Middle	402,424 (51.4%)	216,898 (27.7%)	103,471 (13.2%)	59,796 (7.6%)
High	284,174 (47.3%)	170,836 (28.4%)	88,691 (14.8%)	56,924 (9.5%)

### Number of chronic diseases and all-cause hospitalisations under ambient heat exposure

**Fig. 1:**
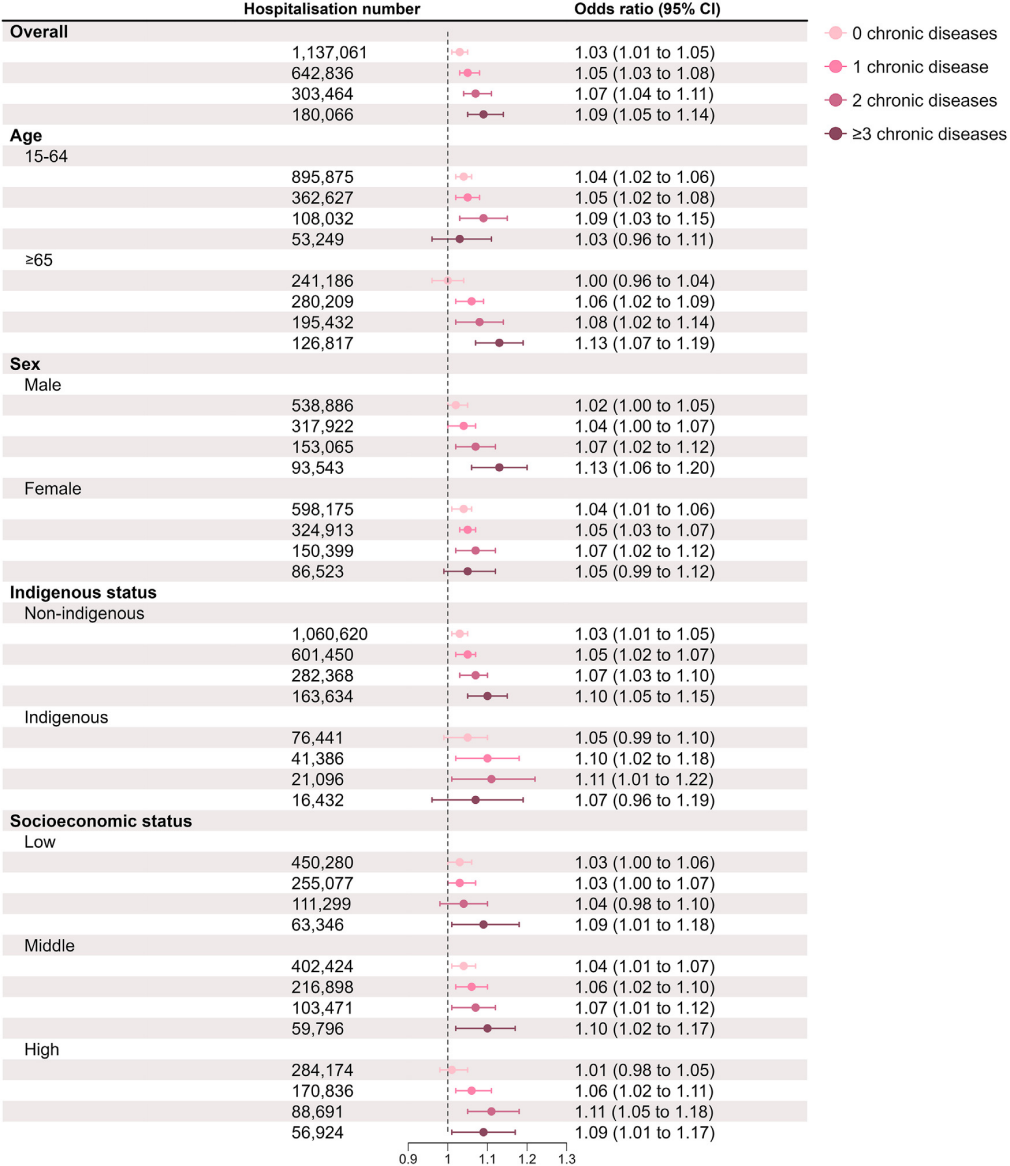
The association between heat exposure and the odds of hospitalisations in people with 0, 1, 2, or ≥3 pre-existing chronic diseases.

Under ambient heat exposure, the odds of all-cause hospitalisations increased with the number of chronic diseases, particularly in older persons, males, and non-indigenous people (Fig. 1) (meta-regression analysis results presented in Table S2 [Appendix]). For instance, in older persons with 0, 1, 2, or ≥3 chronic diseases, the ORs of all-cause hospitalisations associated with 5 °C increase in mean temperature were 1.00 (95% confidence interval [CI]: 0.96, 1.04), 1.06 (1.02, 1.09), 1.08 (1.02, 1.14), and 1.13 (1.07, 1.19), respectively.

### Types and combinations of chronic diseases and all-cause hospitalisations under ambient heat exposure

**Fig. 2:**
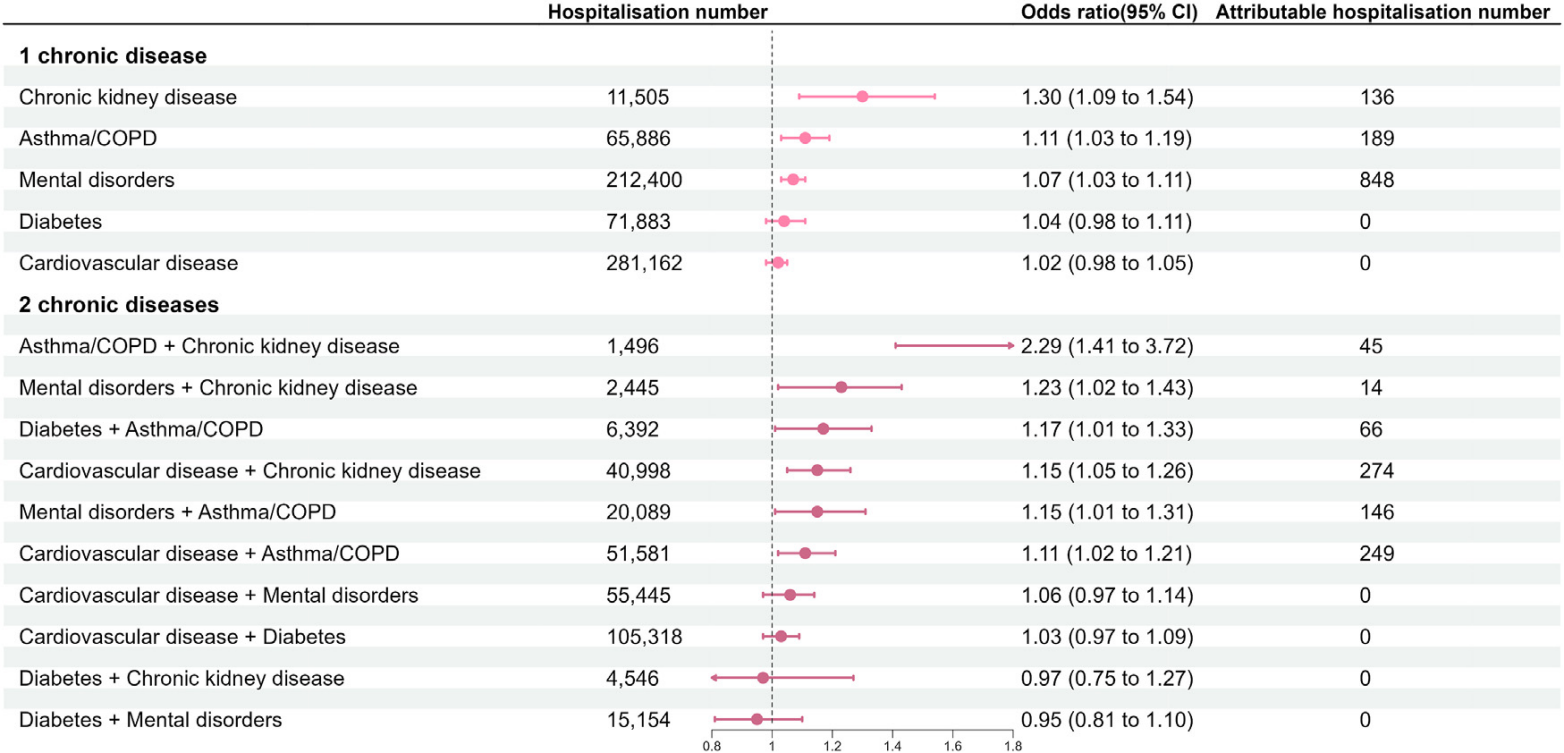
Different types and combinations of pre-existing chronic diseases and the odds and attributable numbers of hospitalisations associated with ambient heat exposure. Attributable hospitalisation number refers to the number of hospitalisations attributable to all temperatures above the reference temperature (25 °C) (i.e., hospitalisations that could have been avoided if exposure to all these temperatures was removed).

Among the groups of chronic diseases, chronic kidney disease, and asthma/COPD, either existing alone (OR: 1.30; 95% CI: 1.09, 1.54, for chronic kidney disease alone; OR: 1.11; 95% CI: 1.03, 1.19, for asthma/COPD alone), together (OR: 2.29; 95% CI: 1.41, 3.72), or in combination with other chronic diseases, were associated with the highest ORs of hospitalisations when mean temperature increased by 5 °C (Fig. 2). The detailed results for all combinations of the five groups of chronic diseases are presented in Figure S2 (Appendix).

Cardiovascular disease in combination with either one, two, or three other groups of chronic diseases was associated with the highest heat-attributable number of hospitalisations, mainly because cardiovascular disease was the most common chronic disease (Figure S2; Appendix).

### Ambient heat exposure and cause-specific hospitalisations in people with multimorbidity

**Fig. 3:**
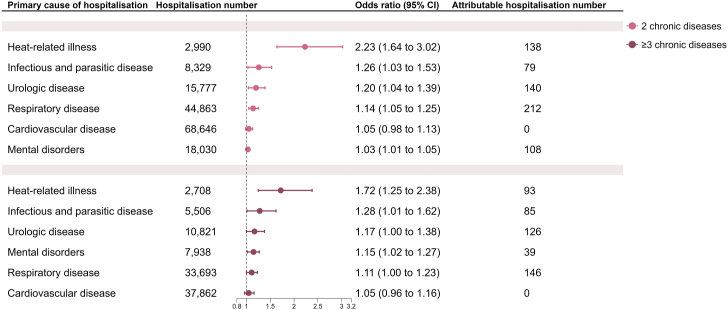
The association between heat exposure and the odds of cause-specific hospitalisations in people with multimorbidity (i.e., 2 or ≥3 pre-existing chronic diseases). Attributable hospitalisation number refers to the number of hospitalisations attributable to all temperatures above the reference temperature (25 °C) (i.e., hospitalisations that could have been avoided if exposure to all these temperatures was removed).

In people with multimorbidity (2 or ≥3 chronic diseases), when the mean temperature increased by 5 °C, their ORs of hospitalisations for heat-related illness, infectious and parasitic disease, and urological disease ranged from 1.17 (95% CI: 1.00, 1.38) to 2.23 (95% CI: 1.64, 3.02) (Fig. 3). When the mean temperature increased by 5 °C, the ORs of hospitalisations for cardiovascular disease, mental disorders, and respiratory disease in people with multimorbidity ranged from 1.03 (95% CI: 1.01, 1.05) to 1.15 (95% CI: 1.02, 1.27).

### Sensitivity analysis results

The results of sensitivity analyses showed that the main results remained robust after considering Parkinson's disease as the sixth group of chronic disease (Figure S3; Appendix), after adjusting the lag period from two days to a week (Figure S4; Appendix), when using maximum temperature as the temperature indicator (Figure S5; Appendix), and when using 1, 2.5, and 7.5 °C as the metric of hot weather (Figures S6, S7, and S8; Appendix).

## Discussion

This study has yielded useful evidence: during hot weather, (1) the odds of all-cause hospitalisations increased with the number of pre-existing chronic diseases; (2) chronic kidney disease and asthma/COPD, either existing alone, together, or in combination with other groups of chronic diseases, were associated with the highest odds of hospitalisations; and (3) the odds of cause-specific hospitalisations, particularly hospitalisations for heat-related illness, infectious and parasitic disease, and urological disease increased substantially in people with multimorbidity.

Several studies in Australia,^33^ China,^39^ Germany,^40^ UK,^41^ and US^42^ examined if certain particular types of pre-existing chronic diseases modified the association between ambient heat exposure and the risk of health events. The Australian, Chinese, and German studies observed that people with diabetes were more likely to have hospitalisations for acute myocardial infarction during hot weather, compared with those without diabetes.^32^^,^^39^^,^^40^ The British study found that the risk of general practitioner consultations for diabetes was elevated during hot weather, particularly for people with cardiovascular or respiratory disease.^41^ The US study reported that hospitalisations and deaths due to end-stage renal disease increased during hot weather, and the elevation in mortality risk was more pronounced in people with COPD, congestive heart failure, or diabetes.^42^ The finding of the present study on chronic disease types and the odds of hospitalisations under ambient heat exposure was in line with these studies, and we further extended the existing knowledge by systematically quantifying the association of chronic disease number, types, and combinations with hospitalisation odds under ambient heat exposure. We believe this evidence provides a more comprehensive picture of chronic diseases (particularly multimorbidity) and heat-health risk.

As a population, females usually have lower height and maximal oxygen consumption compared with males, which could make them biologically more sensitive to ambient heat exposure.^43^ Further, under heat exposure, the body faces hemodynamic challenges for the distribution of blood flow (e.g., perfusing vital organs, maintaining blood pressures, and cutaneous blood flow for heat dissipation). As a consequence of these competing demands on the cardiovascular system, men may be able to call upon a greater cardiac reserve to better maintain thermoregulation via a larger blood volume to circulate^44^ and greater stroke volume for each heartbeat.^45^^,^^46^ A meta-analysis pooling epidemiological evidence also reported a slightly higher risk of mortality associated with heat exposure in females than in males.^47^ However, we observed an association between the number of chronic diseases and the odds of hospitalisations during hot weather in males but not in females. A plausible explanation for this finding is that males are generally involved more in outdoor work and hence exposed more to outdoor hot weather,^48^ making those men with multimorbidity at a higher risk of hospitalisations under ambient heat exposure.

Indigenous people have been reported to be vulnerable to climate change-related extreme weather events,^49^ but empirical evidence on the association between ambient heat exposure and health in indigenous people is scarce.^50^^,^^51^ We observed an association between the number of chronic diseases and the odds of hospitalisations during hot weather in non-indigenous people but not in indigenous people. This finding was somewhat counter-intuitive but in line with a recent study conducted in the Northern Territory, Australia.^17^ Quilty et al. reported that there was a social and cultural adaptation to ambient heat exposure in indigenous people.^17^ Nonetheless, in our study, there was a small sample size in the indigenous people with ≥3 chronic diseases. We believe that the interpretation of the effect estimate in indigenous people with ≥3 chronic diseases needs to be made with caution, because the effect estimate yielded from the small sample could have been less stable. Future larger-sample-size studies examining the association between the number of chronic diseases and heat-health risk in indigenous people are warranted.

### Implications

HHAPs worldwide tend to separately list several chronic diseases that increase people's heat-health risk.^4^ For instance, the Queensland heat-health action plan suggested that pre-existing cardiovascular disease, respiratory disease, and diabetes increase people's heat-health risk, and documented that heat early warnings and support services should be provided to these people during heatwaves.^52^ However, approximately 0.55 million Queenslanders have cardiovascular disease, respiratory disease, or diabetes,^10^ and there is a yearly average of >30 heatwave days in Queensland^53^ (including low-intensity, severe, and extreme heatwaves classified by Excess Heat Factor^54^). It is practically infeasible for the Queensland heat-health action plan to deliver heat early warnings and provide support services to more than half a million people >30 times each summer. A solution for this dilemma is to understand the varying degrees of heat-health risk across segments of the population and prioritise those subgroups most at-risk. Our study provided an example of such attempts. Our findings on chronic disease number and combinations and the odds of hospitalisations under ambient heat exposure suggested that HHAPs should consider specifically examining people with multimorbidity as a distinct heat-vulnerable subgroup and purposefully target these people.

We found that the odds of hospitalisations in people with multimorbidity significantly increased during hot weather. This finding is useful because patients with multimorbidity generally require more comprehensive and integrated medical management. For instance, compared with treating patients with chronic kidney disease alone, treating patients with both mental disorders and chronic kidney disease requires more consideration related to medication adjustments, dietary modification, and fluid intake. For example, some medications used to treat mental disorders could have adverse kidney effects.^55^ Our findings imply that multiple hospital departments are expected to see an appreciable increase in the volume of patients with multimorbidity during hot weather. During medical consultations, clinicians could inform their patients with multimorbidity about their heat-health risk, encourage them to pay more attention to weather forecasting during summer, and avoid non-urgent face-to-face consultations (e.g., consider teleconsultations during hot weather).

### Limitations and strengths

This study has several limitations. First, we restricted the chronic diseases to the five groups of diseases because there is known biological plausibility between these pre-existing chronic diseases and people's heat-health risk. However, this resulted in a relatively small sample size for hospitalisations with ≥3 chronic diseases and wide confidence intervals for the effect estimates (e.g., the confidence intervals in the indigenous people group). Second, due to ethical concerns, we were unable to collect the information on each participant's residential address and therefore were only able to use postcode-level ambient heat exposure as a proxy of their outdoor ambient heat exposure, which could have been subject to exposure measurement bias. Third, we were unable to adjust for humidity in the regression analysis of this study due to the unavailability of postcode-level humidity data in the Australian Bureau of Meteorology online archive.^12^ A recent publication indicated that, for epidemiological studies, it is ideal to explore the influence of humidity on the health outcomes associated with ambient heat exposure using sub-daily climate and health data,^56^ but this was beyond the scope of the present study. Fourth, the duration and severity of chronic diseases (particularly multimorbidity) affect people's health status^57^ and may be associated with their likelihood of hospitalisations during hot weather. However, in our study, we were only able to examine the association between the number of chronic diseases and people's likelihood of hospitalisations during hot weather. Future research is warranted to understand if/how the duration and severity of multimorbidity is associated with people's heat-health risk and to explore effective, accessible, and acceptable strategies for reducing the heat-health risk in people with multimorbidity. Fifth, our study was an observational study, and our findings were observational in its nature and did not imply causality. The magnitude of the effect estimates (e.g., odds ratios around 1.10) indicated that the findings were of statistical significance but not of clinical significance yet.

There are three strengths of this study: (1) the availability of the ‘primary diagnosis and other diagnoses’ ICD-10 codes and the unique ID for each patient allowed us to comprehensively understand the associations of chronic disease number, types, and combinations with the odds of hospitalisations under ambient heat exposure; (2) the statewide data provided a good representative sample of the target population; and (3) a state-of-the-art analytical approach (i.e., the time-stratified case-crossover design with a distributed lag non-linear model) and sensitivity analyses ensured the robustness of the overall findings.

### Conclusions

While individuals with multimorbidity are considered in heat-health action plans, this study suggests the need to consider specifically examining them as a distinct and vulnerable subgroup.

## Contributors

ZX conceptualised the study, conducted the literature search, verified the underlying data, performed the data collection and analysis, and completed the first draft of the manuscript. WY verified the underlying data, performed the data cleaning and analysis, and critically revised the manuscript. AB obtained the funding, conducted the literature search and synthesis, and critically revised the manuscript. ST, KLE, HS, and JC critically revised the manuscript. SR obtained the funding, supervised the project administration, and critically revised the manuscript. All authors read and approved the final version of the manuscript.

## Data sharing statement

Hospitalisation data used in this study were provided by Queensland Health (HlthStat@health.qld.gov.au). Hospitalisation data included ID, date of admission, age, sex, and indigenous status, postcode of residential suburb, primary diagnosis, and other diagnoses. Access to this hospitalisation data requires ethical approval from the Human Research Ethics Committee in Queensland Health. Postcode-level data on maximum and minimum temperature were initially collected by the Australian Bureau of Meteorology and are now publicly available (http://www.bom.gov.au/jsp/awap/temp/archive.jsp?colour=colour&map=maxave&year=2004&month=3&period=daily&area=nat). Postcode-level data on the Index of Relative Socioeconomic Disadvantage were downloaded from the Australian Bureau of Statistics (https://www.abs.gov.au/ausstats/abs@.nsf/mf/2033.0.55.001).

## Declaration of interests

We declare no competing interests.
