# Supplementary material for: Multimorbidity and emergency hospitalisations during hot weather
Source: eBioMedicine. 2024 May 4;104:105148. doi: 10.1016/j.ebiom.2024.105148 (PMC11087953; doi:10.1016/j.ebiom.2024.105148)

**Supplementary appendix**

**Multimorbidity and emergency hospitalisations during hot weather**

Zhiwei Xu, Weizhuo Yi, Aaron Bach, Shilu Tong, Kristie L Ebi, Hong Su, Jian Cheng, Shannon Rutherford

**Caption for supplementary material**

**Figure S1.** The locations of the eight Queensland cities/communities………………………………………. 2

**Table S1.** The five groups of chronic diseases and the corresponding ICD-10 codes……………………….. 3

**Table S2.** The meta-regression analysis results……………………….………………………………………. 4

**Figure S2.** Different types and combinations of pre-existing chronic diseases and the odds and attributable numbers of hospitalisations associated with ambient heat exposure…………………………………………. 5

**Figure S3.** The association between heat exposure and odds of hospitalisations in people with 0, 1, 2, or ≥3 pre-existing chronic diseases (after adding Parkinson’s disease as the sixth group of chronic disease)………….. 6

**Figure S4.** The association between heat exposure and odds of hospitalisations in people with 0, 1, 2, or ≥3 pre-existing chronic diseases (after changing lag period to a week)……………………………………………… 7

**Figure S5.** The association between heat exposure and odds of hospitalisations in people with 0, 1, 2, or ≥3 pre-existing chronic diseases (using maximum temperature as the temperature indicator)………………………. 8

**Figure S6.** The association between heat exposure and odds of hospitalisations in people with 0, 1, 2, or ≥3 pre-existing chronic diseases (using 1 °C increase in mean temperature as the increment)………………………. 9

**Figure S7.** The association between heat exposure and odds of hospitalisations in people with 0, 1, 2, or ≥3 pre-existing chronic diseases (using 2.5 °C increase in mean temperature as the increment)…………………….. 10

**Figure S8.** The association between heat exposure and odds of hospitalisations in people with 0, 1, 2, or ≥3 pre-existing chronic diseases (using 10 °C increase in mean temperature as the increment)……………………... 11

**Figure S1.** The locations of the eight Queensland cities/communities


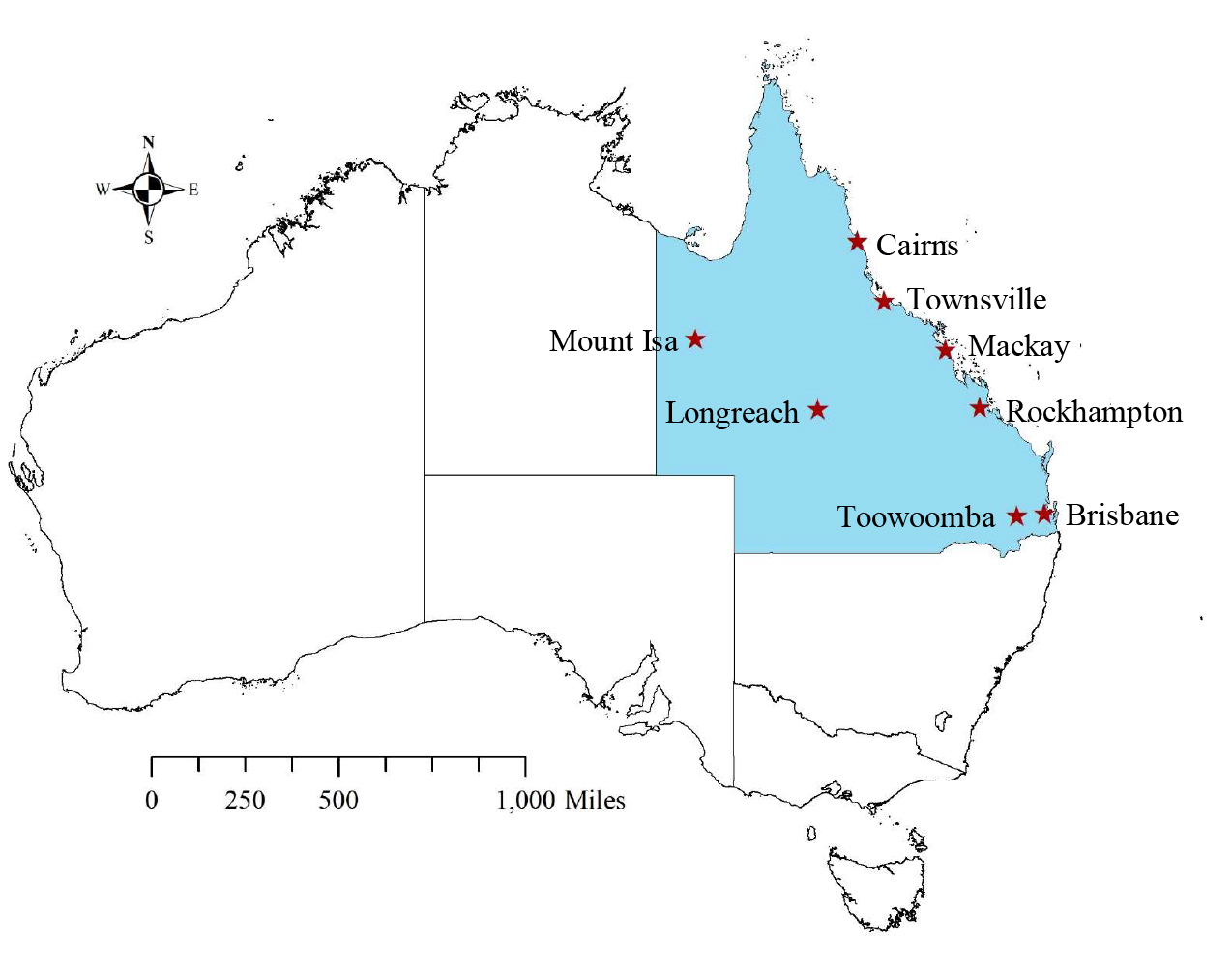


**Table S1.** The five groups of chronic diseases and the corresponding ICD-10 codes

| Disease group | ICD-10 codes |
| --- | --- |
| **Cardiovascular disease** |  |
| hypertensive heart disease | I10-I15 |
| ischaemic heart disease | I20-I25 |
| heart failure | I50 |
| stroke | I60-I69 |
| **Diabetes** | E10-E14 |
| **Mental disorders** |  |
| schizophrenia, schizotypal and delusional disorders | F20-F29 |
| bipolar disorders | F30, F31, F34.0 |
| depressive disorders | F32, F33, F34.1, F34.8-F39 |
| anxiety disorders | F40-F43 |
| dementia, including Alzheimer’s disease | F00, F01, F03, G30 |
| **Asthma/COPD** | J45 for asthma and J41-J44 for COPD |
| **Chronic kidney disease** | N18 |

**Table S2.** The meta-regression analysis results showing the association between chronic disease number and the likelihood of hospitalisations during hot weather

|  | β | P value |
| --- | --- | --- |
| **Overall** | 0.0189 | 0.0043 |
| **Age** |  |  |
| 15-64 | 0.0079 | 0.4073 |
| ≥65 | 0.0389 | 0.0003 |
| **Sex** |  |  |
| Male | 0.0295 | 0.0015 |
| Female | 0.0077 | 0.4010 |
| **Indigenous status** |  |  |
| Non-indigenous | 0.0207 | 0.0028 |
| Indigenous | 0.0138 | 0.4424 |
| **Socioeconomic status** |  |  |
| Low | 0.0120 | 0.2896 |
| Middle | 0.0171 | 0.1042 |
| High | 0.0336 | 0.0045 |

**Figure S2.** Different types and combinations of pre-existing chronic diseases and the odds and attributable numbers of hospitalisations associated with ambient heat exposure. Attributable hospitalisation number refers to the number of hospitalisations attributable to all temperatures above the reference temperature (25 °C) (i.e., hospitalisations that could have been avoided if exposure to all these temperatures was removed)


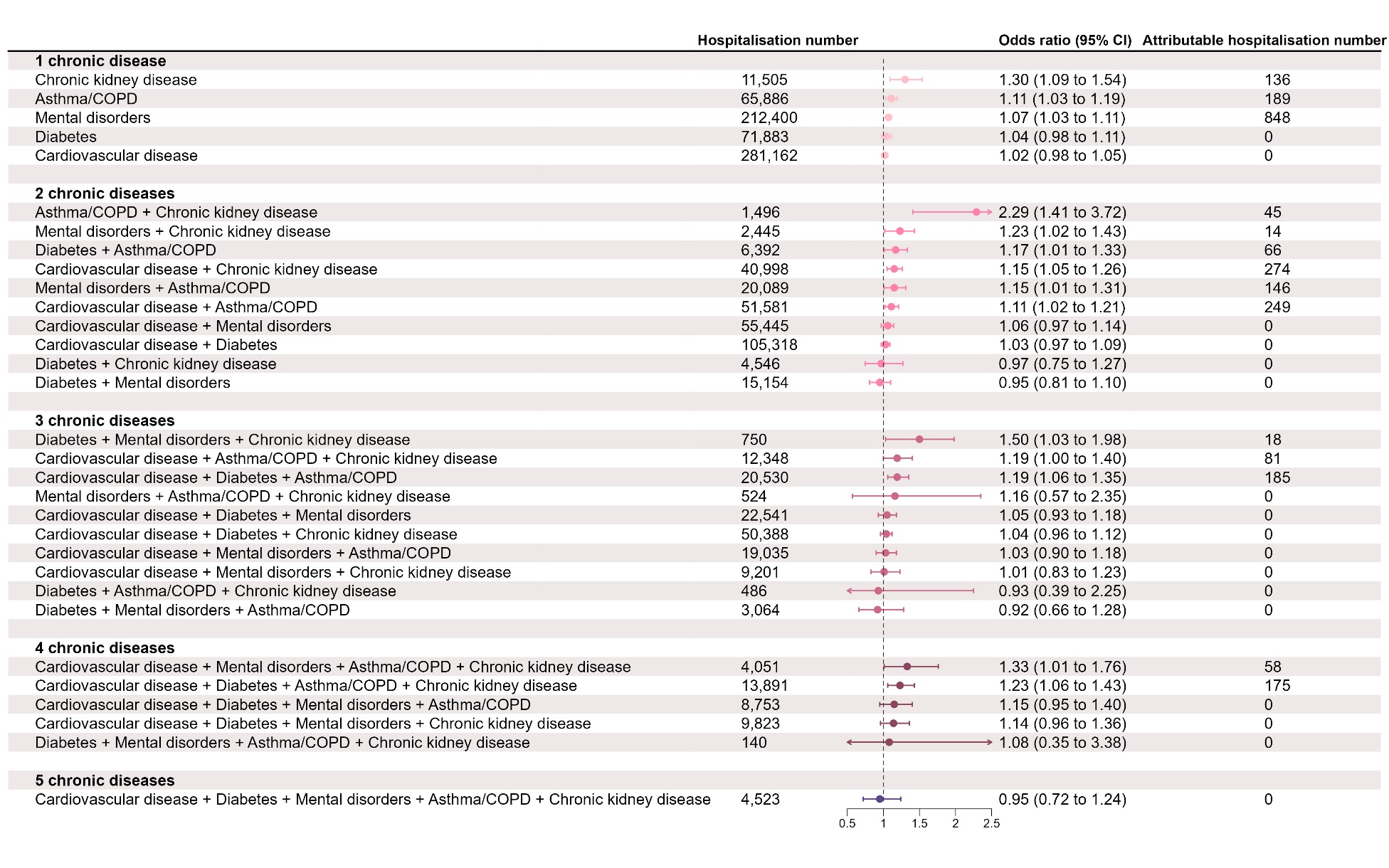


**Figure S3.** The association between heat exposure and odds of hospitalisations in people with 0, 1, 2, or ≥3 pre-existing chronic diseases (after adding Parkinson’s disease as the sixth group of chronic disease)


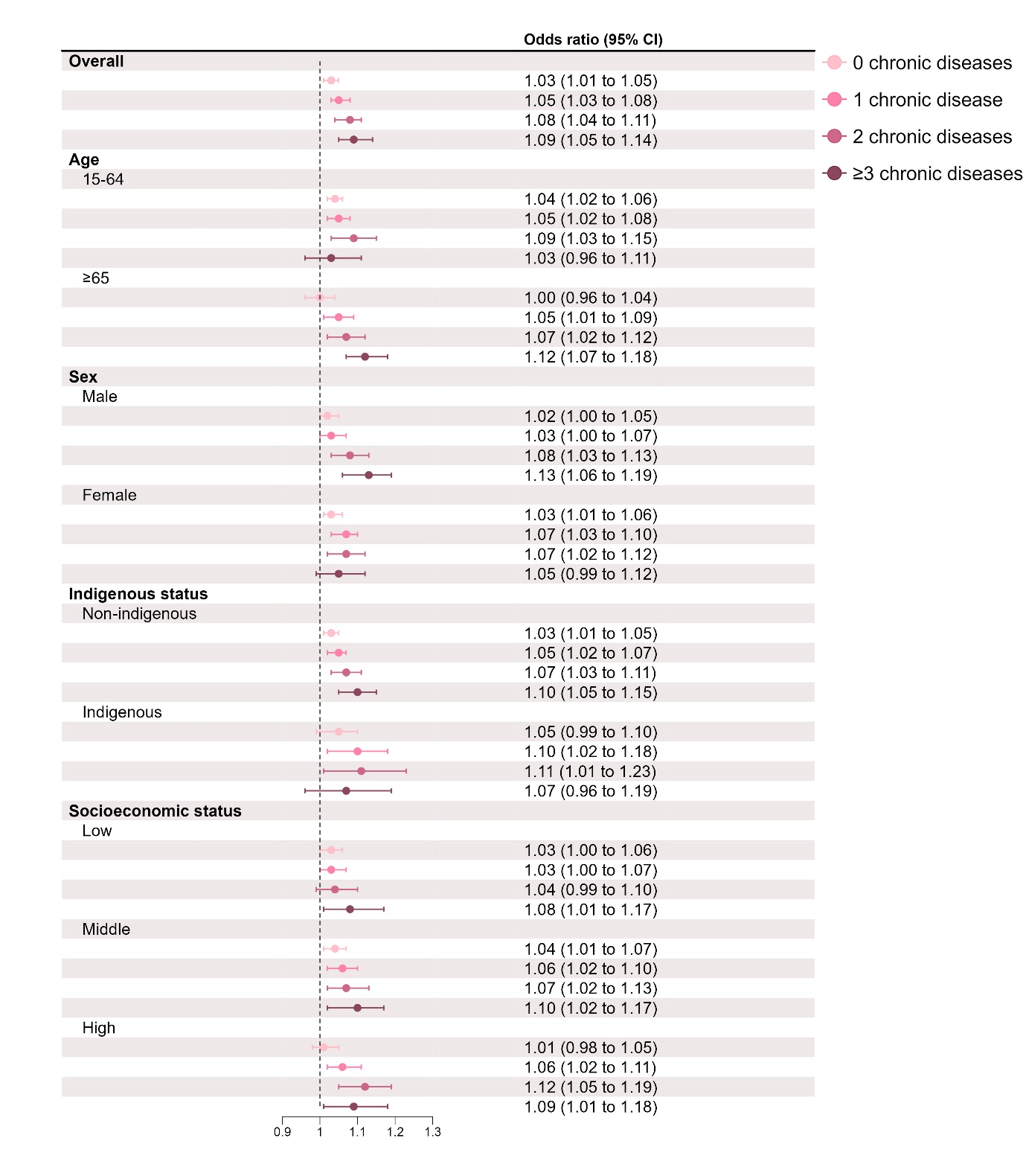


**Figure S4.** The association between heat exposure and odds of hospitalisations in people with 0, 1, 2, or ≥3 pre-existing chronic diseases (after changing lag period to a week)


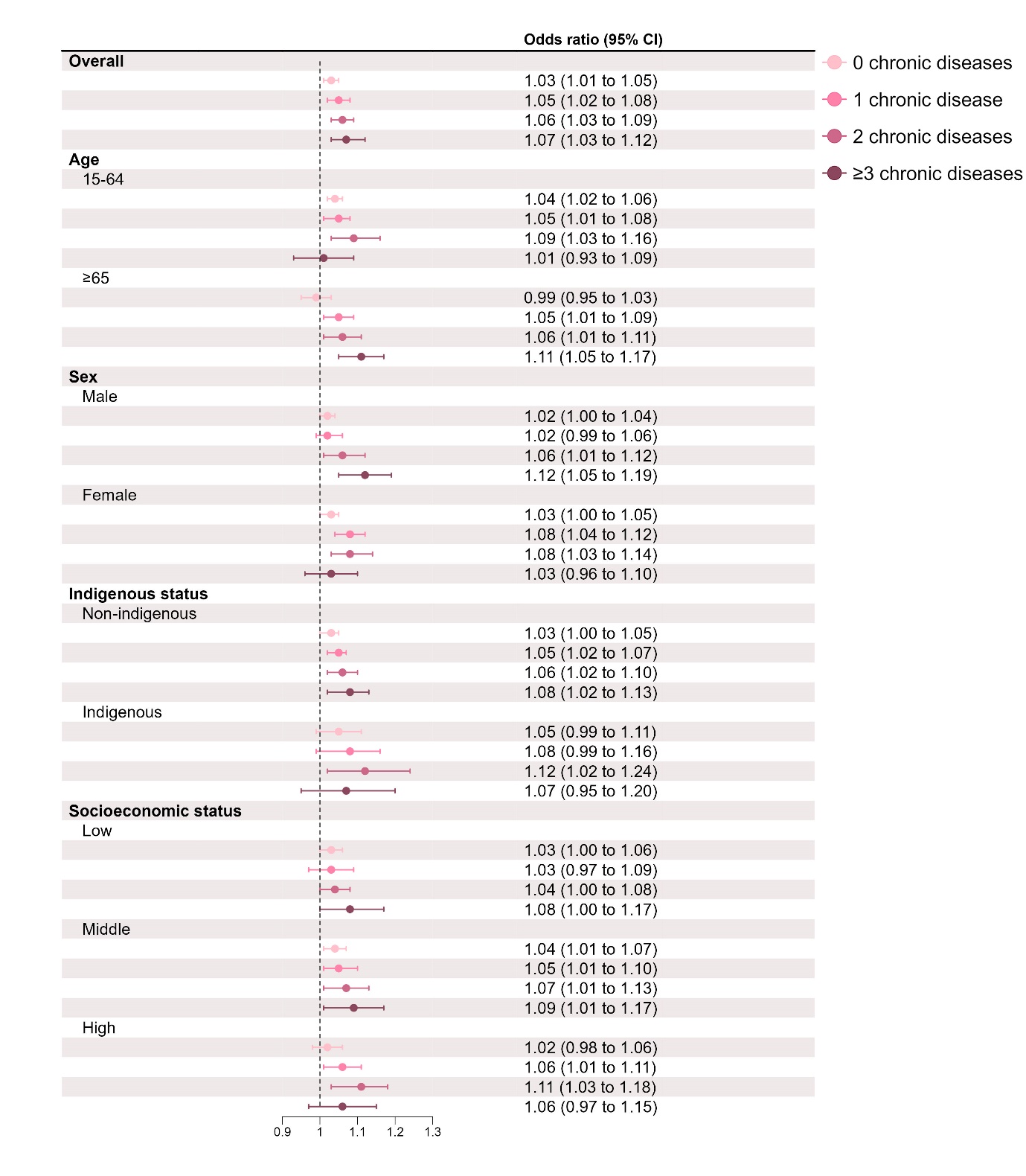


**Figure S5.** The association between heat exposure and odds of hospitalisations in people with 0, 1, 2, or ≥3 pre-existing chronic diseases (using maximum temperature as the temperature indicator)


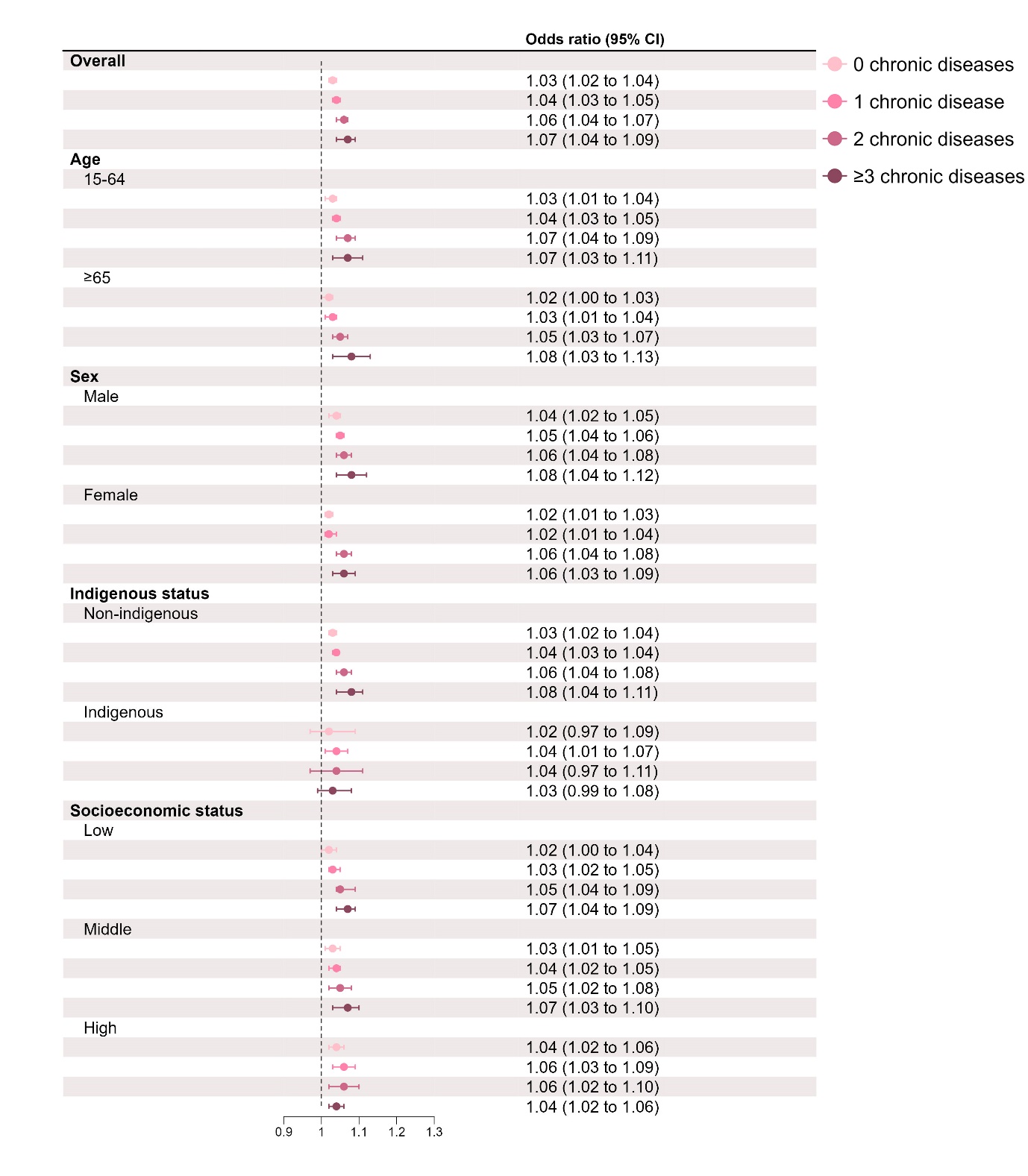


**Figure S6.** The association between heat exposure and odds of hospitalisations in people with 0, 1, 2, or ≥3 pre-existing chronic diseases (using 1 °C increase in mean temperature as the increment)


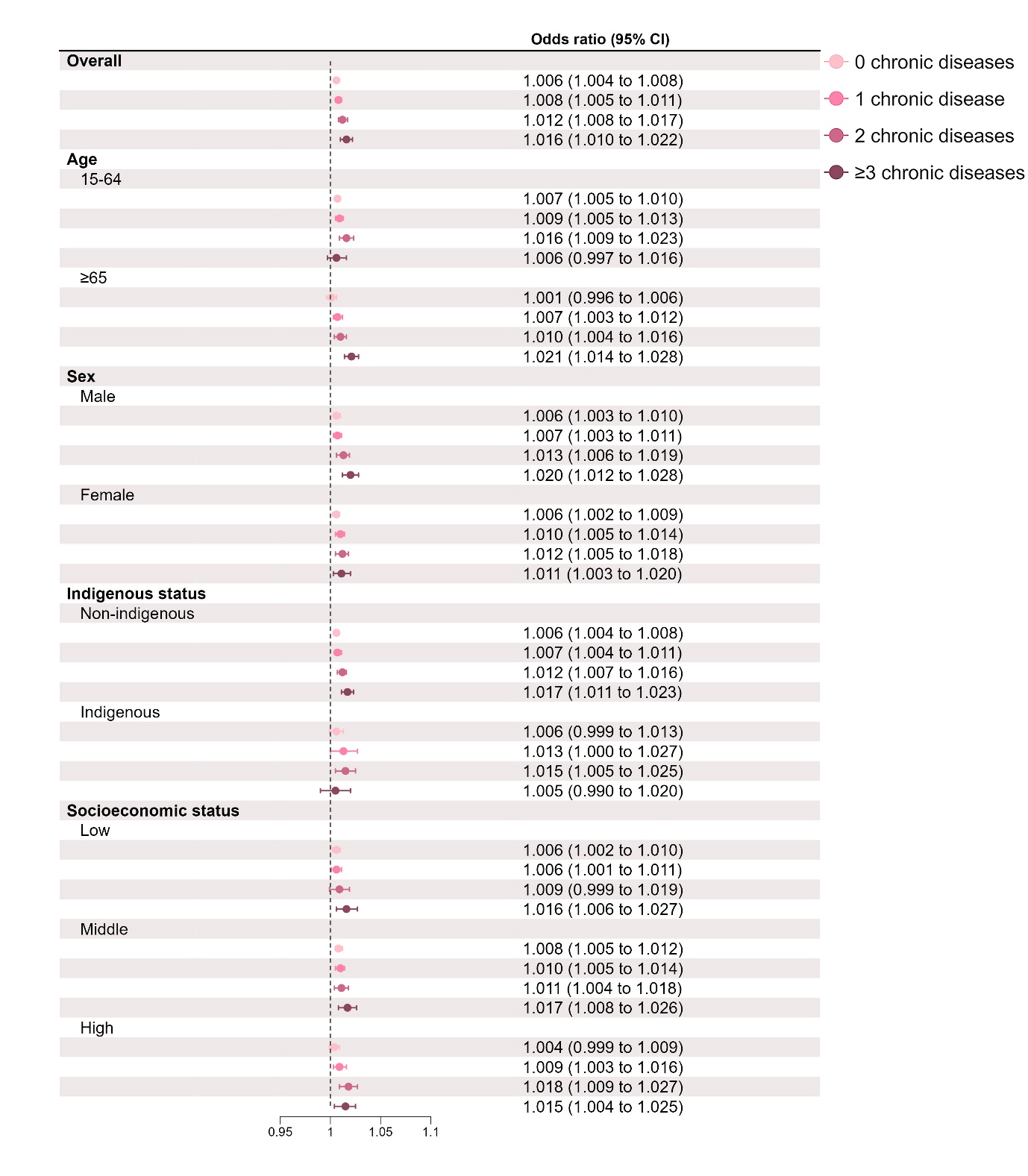


**Figure S7.** The association between heat exposure and odds of hospitalisations in people with 0, 1, 2, or ≥3 pre-existing chronic diseases (using 2.5 °C increase in mean temperature as the increment)


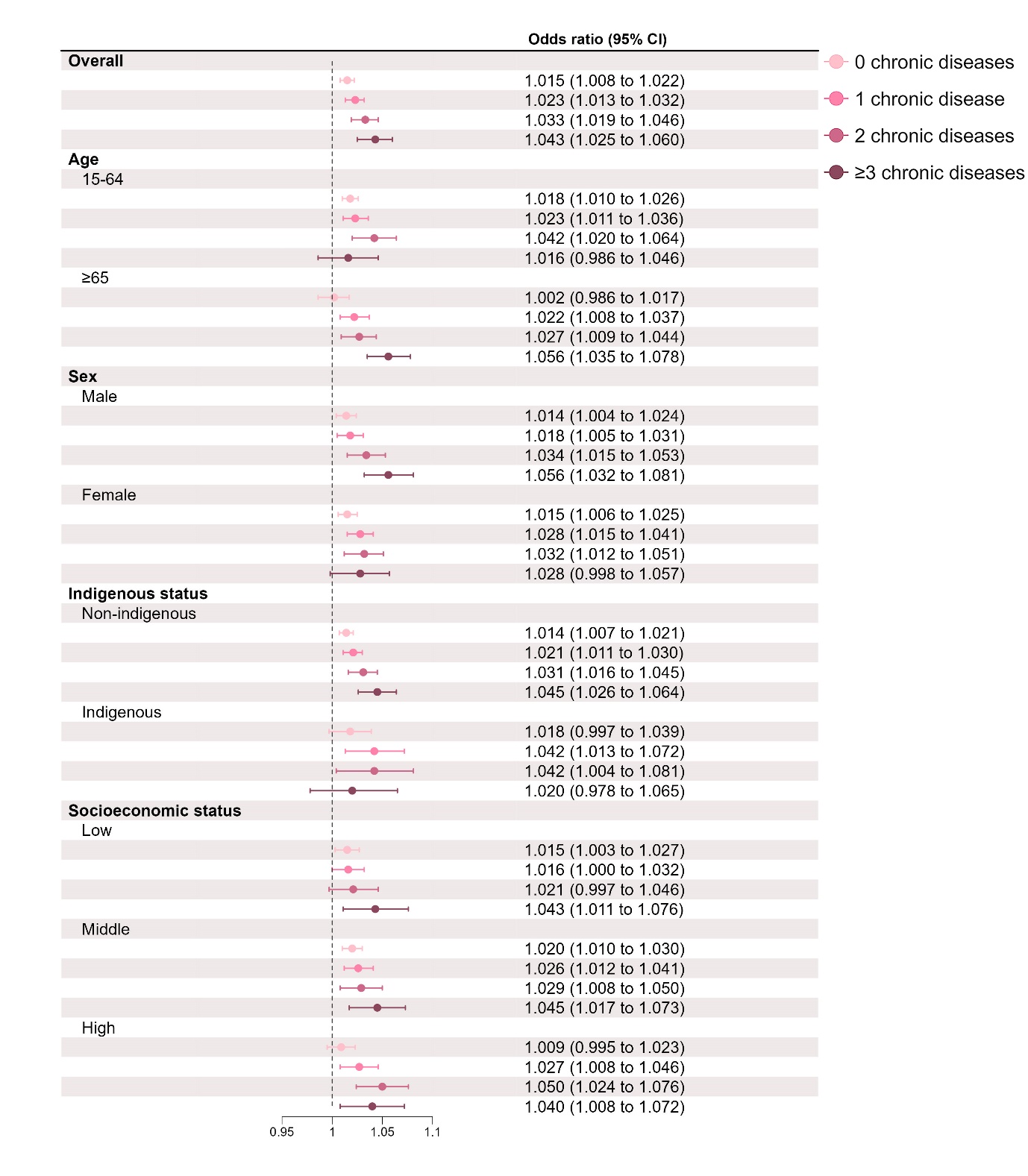


**Figure S8.** The association between heat exposure and odds of hospitalisations in people with 0, 1, 2, or ≥3 pre-existing chronic diseases (using 7.5 °C increase in mean temperature as the increment). Results for individuals with ‘low’ or ‘high’ socioeconomic status were not available because there were not enough number of days with mean temperature >32.5 °C when these individuals were admitted to hospitals


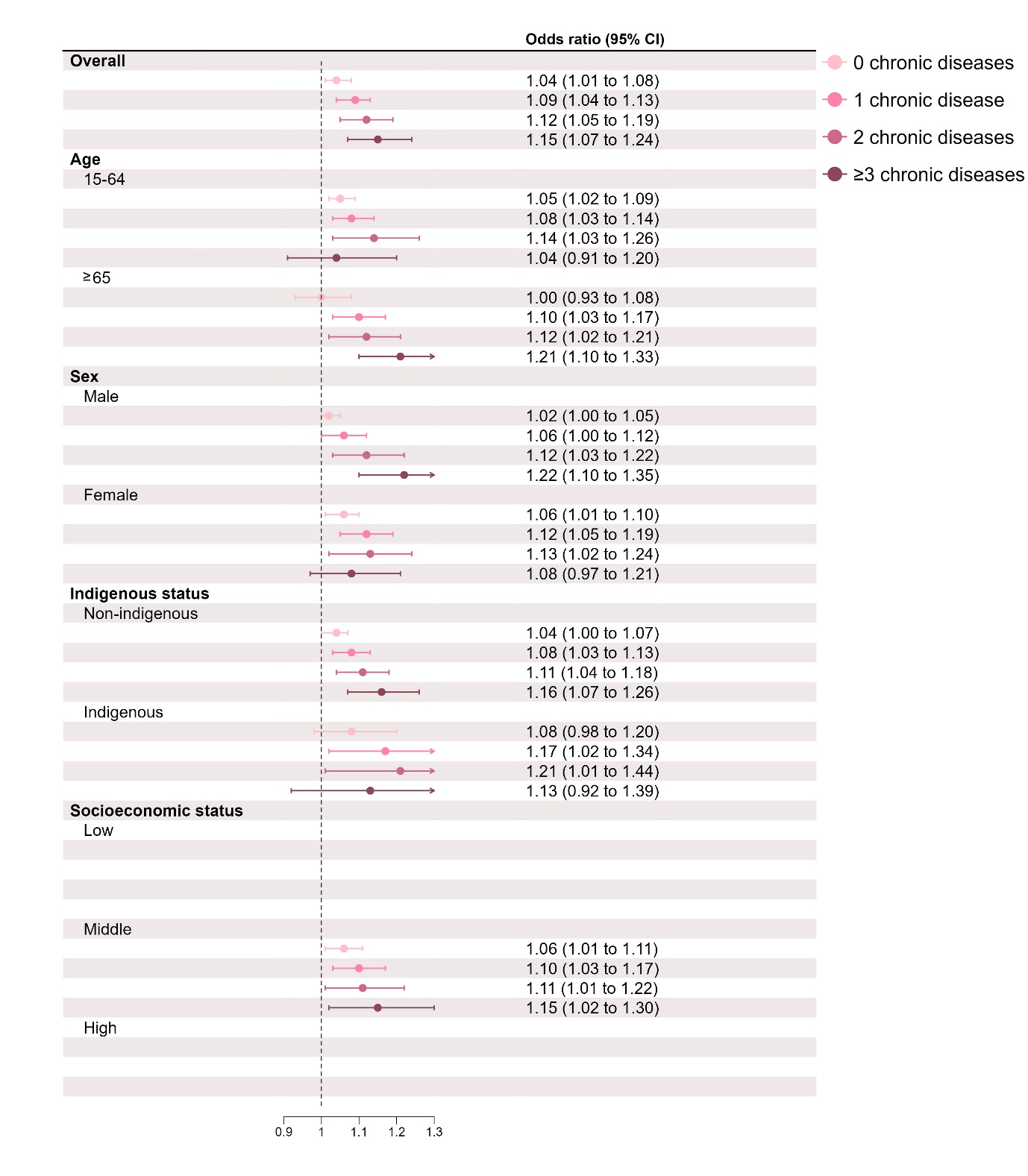

Supplement: Figures and Tables [file mmc1.docx]
